# Functional Analysis of *Drosophila* HSP70 Promoter with Different HSE Numbers in Human Cells

**DOI:** 10.1371/journal.pone.0101994

**Published:** 2014-08-07

**Authors:** Nadezda Kust, Ekaterina Rybalkina, Ilya Mertsalov, Ekaterina Savchenko, Alexander Revishchin, Gali Pavlova

**Affiliations:** 1 Institute of Gene Biology, Russian Academy of Sciences, Moscow, Russia; 2 Institute of Medicine and Cell Transplantation, Moscow, Russia; 3 Ltd Apto-pharm, Moscow, Russia; University of Arkansas for Medical Sciences, United States of America

## Abstract

The activation of genetic constructs including the *Drosophila hsp70* promoter with four and eight HSE sequences in the regulatory region has been described in human cells. The promoter was shown to be induced at lower temperatures compared to the human *hsp70* promoter. The promoter activity increased after a 60-min heat shock already at 38°C in human cells. The promoter activation was observed 24 h after heat shock for the constructs with eight HSEs, while those with four HSEs required 48 h. After transplantation of *in vitro* heat-shocked transfected cells, the promoter activity could be maintained for 3 days with a gradual decline. The promoter activation was confirmed *in vivo* without preliminary heat shock in mouse ischemic brain foci. Controlled expression of the *Gdnf* gene under a *Drosophila hsp70* promoter was demonstrated. This promoter with four and eight HSE sequences in the regulatory region can be proposed as a regulated promoter in genetic therapeutic systems.

## Introduction

Gene and cell therapy opens up new opportunities for treating neurological diseases. However, despite considerable advances in this field, the progress in clinical practice is still limited. Among other things, this is due to insufficient choice of promoters for therapeutic genes. The downside of gene therapeutic systems is insufficient regulation of gene expression, which makes it difficult to maintain their expression at the level required for the healing effect. Widely used cytomegalovirus (CMV) promoter proved to have highly variable activity in different tissues [1]. It is nearly inactive in undifferentiated mammalian embryonic cells [2]. Application of ubiquitin promoters does not allow transgene expression to be limited to desired cell population or tissue. Ectopic expression of exogenous genes can have dangerous side effects including inflammation and immune responses [3]. Inducing agent toxicity as well as the immune response to chimeric transactivators in the case of transcription factors activated by small molecules (e.g., tetracycline-activated systems) prevents their clinical application [4], [5], [6]. This substantiates the discovery of new regulated promoters.

A regulated promoter activated without inducing agents can be exemplified by the heat shock protein promoter. The *hsp70* promoter is activated by increased temperature alone. However, the mammalian *hsp70* promoter is induced by body temperature increase to 42°C, which is traumatic for mammalian cells. The *hsp70* promoter in *Drosophila melanogaster* is activated in insect cells at 37°C. System with such properties could be highly convenient in cell therapy since human/mammalian body temperature can directly stimulate the *Drosophila* heat shock protein promoter [7]. Previously, we have shown that the introduction of the *Drosophila melanogaster hsp70* promoter into mammalian cells changes its activation temperature to 39–40°C, which is higher than the normal mammalian body temperature (37°C) but lower than the activation temperature of the mammalian *hsp70* promoter (42°C) and further from the upper limit of the physiological temperature range [8]. Apart from high temperature, *hsp70* promoter can be activated by a variety of other stress stimuli including hypoxia [9], ischemia [10], and infection [11]. In this context, a therapeutic gene can be activated in transgenic cells injected into an ischemic focus or other pathological area with inflammation. Thus, further studies of the regulatory regions of *hsp70* promoter are relevant to elucidate the mechanisms underlying its regulation and to apply this promoter in the development of gene therapeutic constructs.

The *Drosophila hsp70* promoter can be activated in mouse and monkey cells as well as in Xenopus oocytes [12]. The promoter activation is mediated by the interaction between the heat shock factor (HSF) and heat shock element (HSE) [13]. The prepromoter region of the *Drosophila hsp70* gene contains four HSEs, each of which includes three or four 5-bp sequences 5′-NGAAN-3′ [14], [15]. The *Drosophila hsp70* promoter can also be heat shock-activated in yeasts, mouse cells, monkey COS cells, and Xenopus oocytes [12]. Yeast transfection by the construct with β-galactosidase gene under control of the *Drosophila hsp70* promoter with different modifications of the regulatory region demonstrated that stable promoter activation requires two or more sequences composed of 5′-NGAAN-3′ and complementary 5′-NTTCN-3′ pentamers. The promoter inducibility increases with the number of such sequences [16]. Such strong cooperativity of binding HSEs by HSF assumes that the heat shock gene expression depends both on the length and number of HSEs [17].

The present work studied the functional properties of the *Drosophila hsp70* promoter with different numbers of HSEs (four and eight) in the regulatory region in human cells. The temperature range as well as the effect of HSEs in the regulatory region on the promoter activation were determined. After transplantation of *in vitro* heat-shocked transfected cells into the mouse brain, the promoter activity could be maintained for 3 days with a gradual decline. The promoter activation *in vivo* without preliminary heat shock was confirmed in mouse ischemic brain foci. Controlled expression of the GDNF gene under a *Drosophila hsp70* promoter was demonstrated.

## Materials and Methods

### Plasmid Constructs

**Figure 1:**
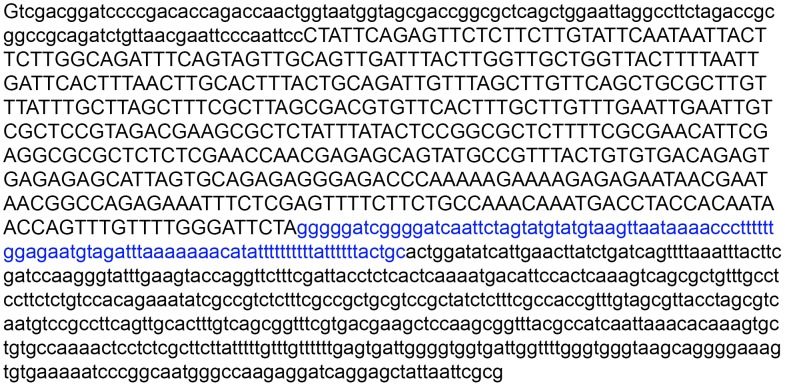
Nucleotide sequence of the hsp70+500 bp fragment cloned into pGem-T Easy. Capital letters indicate the *hsp70* promoter sequence (453 bp). The 100 bp regulatory sequence included in the 500 bp regulatory region is shown in italics. Sequencing was performed using the reverse primer phsR (5^′^-taacgaattcccaattccctattcag-3′).

**Figure 2:**
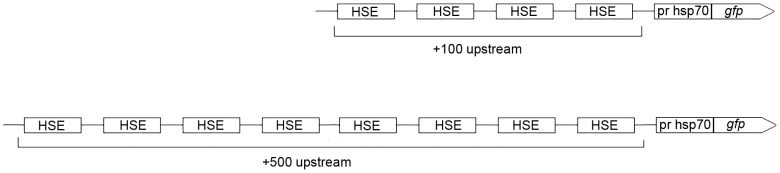
Diagram of heat shock elements (HSEs) in the regulatory region of generated factors B7 (upper) and F7 (lower).

Two vectors B7 and F7 (Figs. 1, 2) were generated to study the regulated promoter of the *Drosophila melanogaster hsp70* gene. All constructs were based on pEGFP-N1 (GenBank Acc. # U55762) and pCaSpeR-hs/act (GenBank Acc. # U60735) vectors. The HindIII/EcoRI fragments (553 and 953 bp) containing the *hsp* promoter (453 bp) and the regulatory region of 100 bp (four HSEs) (B7) or 500 bp (eight HSEs) (F7) were used for the study. The fragments were generated by PCR from the pCaSpeR-hs/act plasmid using the following primers: Casp^1^F, 5′-gatcaagcttgttcaatgatatccagtgc-3^′^; Casp^5^F, 5^′^-cccaaagcttggatttttcacactttcccc-3^′^; and CaspR, 5^′^-taacgaattcccaattccctattcag-3^′^. Casp^1^ and Casp^5^ were generated by pCaSpeR-hs/act digestion with EcoRI. The resulting fragments were sequenced to confirm their authenticity and cloned into EcoRI-digested pGEM-T Easy vector (Promega #1360) following the manufacturer's protocol. The resulting constructs pGEM/Casp^1^ and pGEM/Casp^5^ were digested with HindIII and EcoRI and the 553 and 953 bp fragments were cloned into the corresponding sites of the EGFP-N1 vector, respectively. The authenticity of the F7(pEGFP-N/Casp^1^) and F7(pEGFP-N1/Casp^5^) vectors was confirmed by restriction endonuclease analysis and sequencing. These vectors contain the *gfp* gene as a marker and the neomycin resistance gene, which allows G418-selection of the transfectants.

The B7 and F7 vectors were used to engineer two constructs containing the glial cell line-derived neurotrophic factor (*Gdnf*) gene. Human *Gdnf* gene was chemically synthesized using the sequence from the UniProtKB (first isoform of P39905) (Evrogene). The *Gdnf* gene fragment of 650 bp was amplified by PCR using primers T3(F) 5^′^-attaaccctcactaaaggga-3^′^ and *Gdnf* ^BamH1^ 5^′^-tggatcccagatacaccacaccttttagcgg-3^′^ and cloned into the pGEM-T Easy vector (Promega #1360) following the manufacturer's protocol. The authenticity of the resulting pGEM/*Gdnf* construct was confirmed by EcoRI endonuclease analysis and sequencing. The 600 bp EcoRV/BamHI fragment of pGEM/*Gdnf* containing the *Gdnf* gene was cloned into the EcoRV and SmaI sites of B7 and F7 using the Klenow fragment (Fermentas). The authenticity of the resulting constructs B7/*Gdnf/gfp* and F7/*Gdnf/gfp* was confirmed by restriction endonuclease analysis and sequencing.

### Cell Cultures and Transfection

Cells HEK293 were cultured in 25 cm^2^ flasks (Costar) with DMEM (PanEko) supplemented with 10% fetal calf serum (Perbio HyClone), 4 mg/ml L-glutamine (PanEko), and 4% gentamicin (PanEko) in a CO_2_ incubator at 37°C and 5% CO_2_. The cells (1.5×10^5^ cells/ml) were transfected with the B7 or F7 vectors using ExGene 500 (Termo Scientific, R0511). The transfected clones were selected with 0.4 mg/mm geneticine (G418, Invitrogen # 15750045) for 10 days, after which he RNA was isolated from G418-resistant clones for RT-PCR analysis. As a result, the following transgenic cell cultures were established: HEK293/B7/*gfp*, HEK293/F7/*gfp*, HEK293/B7/*Gdnf/gfp*, and HEK293/F7/*Gdnf/gfp*.

### Reverse Transcription Polymerase Chain Reaction

Cultured cells were harvested from flasks using trypsin and Versene (PanEko). RNA isolated from transgenic cell cultures was used for RT-PCR. RNA was isolated using Trisol reagent (Sigma). Reverse transcription was performed in the reaction mixture containing 5 µg RNA, 0.5 µg oligo(dT), and 20 µl H20 using a BioRad amplifier. cDNA was synthesized from single-stranded RNA using a RevertAid Reverse Transcriptase (M-MuLV RT) (Termo Scientific) using the following program: 70°C, 5 min; 37°C, 60 min; 70°C, 5 min; and 20°C, 30 s. The synthesized cDNA was used in PCR with the following primer pairs: neo(R) 5^′^-tcagaagaactcgtcaagaa-3^′^ and neo(F) 5^′^-atgattgaacaagatggatt-3^′^, 795 bp PCR product; GAPDH(R) 5^′^-cccctggccaaggtcatccatgacaactt-3^′^ and GAPDH(F) 5^′^-ggccatgaggtccaccaccctgttgctgta-3^′^, 513 bp product; *Gdnf*(F) 5^′^-ggaatcggcaggctgcagctg-3^′^ and *gfp*(R) 5^′^-aataaagcttgcatggcggtaatacg-3^′^, 1211 bp product; *gfp*(F) 5^′^-cgtcagatccgctagcgctaccgg-3^′^ and *gfp*(R) 5^′^- aataaagcttgcatggcggtaatacg-3^′^, 715 bp product. The PCR program consisted of 94°C for 2 min; 30 cycles of 93°C for 10 s, 58°C for 20 s, and 72°C for 30 s; and final 72°C for 10 min.

### In vitro Promoter Activation

Promoter activation was tested in transgenic cells HEK293/B7/*gfp* and HEK293/F7/*gfp* after heat shock. The cells were plated on 30 mm dishes at 70–80% confluency, exposed to temperatures from 37 to 42°C for 60 min, and placed back to normal culture conditions. GFP expression was examined 24 and 48 h after heat shock under an Olympus IX81 microscope or using a FACScan flow cytometer (Becton Dickinson) in green FL1 channel. In the latter case cells were rinsed twice with PBS and fixed with 2% formaldehyde in PBS at 4°C for 20 min. Four days after heat shock, a fraction of cells was exposed to heat shock again. Promoter activation in transgenic cells HEK293/B7/*Gdnf/gfp* and HEK293/F7/*Gdnf/gfp* was tested in a similar way.

### Behavior of Transfected Cells Injected into Mouse Brain

Transgenic cells HEK293/B7*/gfp* and HEK293/F7*/gfp* as well as HEK293/B7/*Gdnf/gfp*, and HEK293/F7/*Gdnf/gfp* were injected into the striatum of Black-6 mice. Animals anesthetized by chloral hydrate were placed in a stereotaxic frame. A suspension containing about 100,000 cells in 1 µl of Hanks solution was gradually injected with a microsyringe into the right cerebral hemisphere at coordinates AP 0 mm and ML 2.5 mm. Transgenic cells HEK293/B7/*gfp*, and HEK293/F7/*gfp* were injected after pre-injection of 1 µg of endothelin-1 in 1 µl saline for 10 min into the same site. The needle was inserted to a depth of 2.5 mm and withdrawn in steps to a depth of 1.5 mm. After 3–5 days, the animals were anesthetized again and perfused through the heart with PBS and then with 4% formaldehyde in PBS. The brain was isolated, fixed again in formaldehyde for 12 h at 4°C, and soaked in 30% sucrose in PBS. The cryotome coronal sections of the brain (40 µm) were analyzed using an Olympus microscope IX81 optimized for GFP visualization. Cells were counted using the Cell^P^ software (Olympus Soft Imaging Solution GmbH).

### Statistical Analysis

Data are presented as SEM ± SD. The statistical analysis was performed using the SPSS software. GFP-positive cell counts were averaged for each group and compared by one-way ANOVA followed by Tukey's multiple comparisons test. Statistical significance was accepted at P<0.05.

The study was approved by the Ethics Committee of Moscow State University (Permit Number 24-01). It was carried out in strict accordance with the ethical principles and scientific standards of the Ethics Committee of Moscow State University.

## Results

### Generation of Genetic Constructs

Regulated *Drosophila melanogaster hsp70* promoter induced by heat shock was used in this work. Two vectors with different regulatory regions of the promoter were synthesized. One of them (B7) includes 100 bp (with four regulatory HSE sequences) upstream of the promoter, and the other vector (F7) includes 500 bp (with eight HSEs). Different amounts of the transcription factor HSF required for the promoter activation can be expected to bind the regulatory regions of these factors. The vectors contain the *gfp* gene as an expression marker and the neomycin resistance gene, which allows G418-selection of the transfectants. The HindIII-EcoRI fragments containing the hsp70 promoter and the regulatory region with the 100 and 500 bp sequences were isolated from the pCasper-hs-act vector. The subfragments amplified by PCR using specific primers hsR (5^′^-taacgaattcccaattccctattcag-3′), phs1F (5^′^-gatcaagcttgttcaatgatatccagtgc-3′), and phs5F (5^′^-cccaaagcttggatttttcacactttcccc-3′) were cloned into pGEM-T Easy (Promega, # 1360). HindIII-EcoRI fragments ‘hsp70+100 upstream’ and ‘hsp70+500 upstream’ were isolated from the resulting vectors, respectively (Fig. 1).

### Constructs Containing *gfp* Gene under Control of Drosophila *hsp70* Promoter

**Figure 3:**
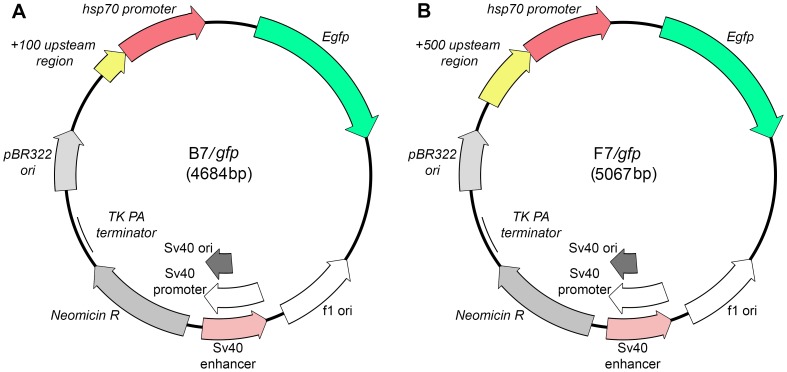
Diagram of genetic constructs containing the *gfp* gene under control of the *hsp70* promoter with the transgenic regulatory region of (A) 100 bp (B7) or (B) 500 bp (F7).

These fragments were cloned into the HindIII and EcoRI sites of the pEGFP-N1 vector. The authenticity of these constructs was confirmed by restriction endonuclease analysis and sequencing. The resulting vectors B7 and F7 contained the *Drosophila hsp70* promoter, polylinker sequence, and *gfp* gene as a visual marker (Fig. 2, 3). B7 contains four HSEs in the promoter regulatory region, while F7 has eight HSE sequences (Fig. 2).

Isolated and purified B7 and F7 were introduced into HEK293 cells by transfection using ExGene 500 (Fermentas, R0511). PCR analysis of the total DNA isolated from HEK293 transgenic cells has confirmed the presence of the *gfp* gene in their genome. PCR was performed using primers *gfp*F (5^′^-cgtcagatccgctagcgctaccgg-3^′^) and *gfp*R (5^′^-aataaagcttgcatggcggtaatacg-3^′^) and the following profile: 94°C for 2 min; 30 cycles of 93°C for 10 s, 58°C for 20 s, and 72°C for 30 s; and final 72°C for 10 min.

**Figure 4:**
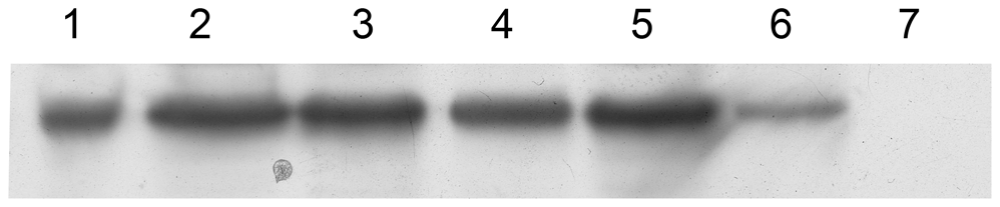
Western blot analysis of HEK293 cells exposed to heat shock at 40°C using antibodies against GFP (Rabbit/polyclonal Abcam ab 290). 1, proteins isolated from HEK293 cells with *gfp* under control of the CMV promoter; 2–4, proteins isolated from HEK293/B7 cells; 5–6, proteins isolated from HEK293/F7 cells; 7, proteins isolated from HEK293 cells.

Proteins isolated from the transgenic cultures were separated by SDS-PAGE (10%) and analyzed by Western blot. Rabbit polyclonal anti-GFP antibodies and secondary horseradish peroxidase-conjugated anti-rabbit secondary antibodies were used. Peroxidase complexes were detected using an ECL reagent. Thus, the constructs presence in cells has been confirmed (Fig. 4).

After G418-selection for 10 days and 24 h incubation in full medium, transfected cells were exposed to heat shock at temperatures from 38 to 42°C for 60 min. Control cells were not exposed to heat shock.

**Figure 5:**
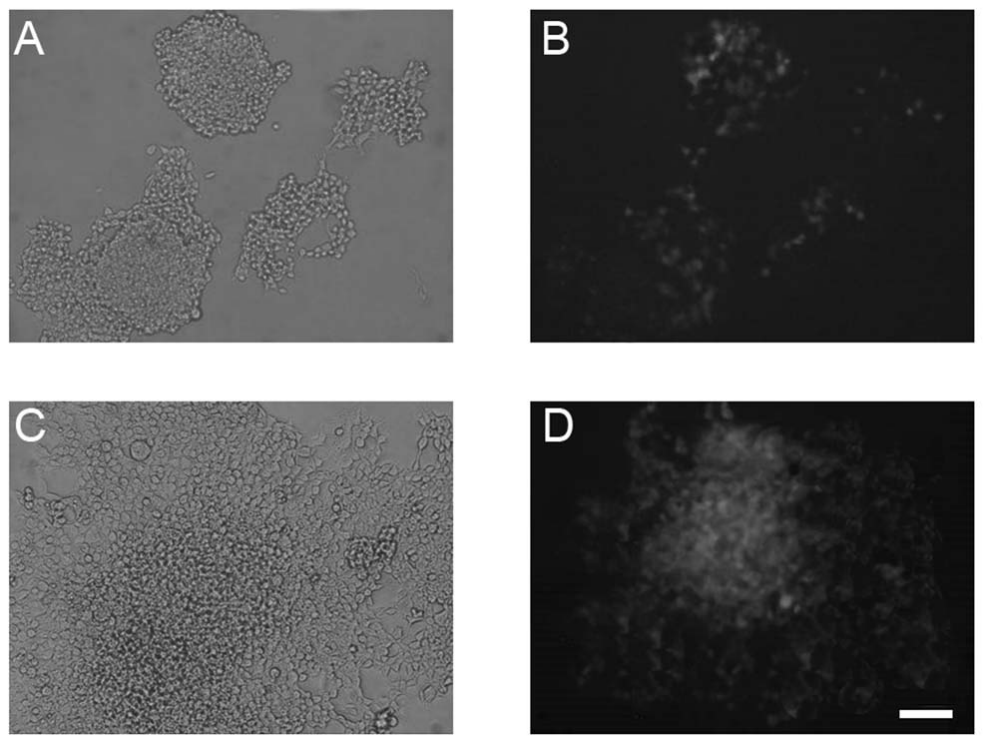
HEK293 cells after transfection with vectors B7 (A, B) and F7 (C, D). A micrographs of cell culture 48h after heat shock at 42°C made in phase contrast (A, C) and GFP fluorescence (B, D). Scale bar on D–200 µm.

Fluorescent microscopy indicated a small number (up to 7%) of cells expressing the marker protein in transfected cells. Twenty-four hours after heat shock at 42°C, the culture transfected with the F7 vector (eight HSEs + hsp70 promoter) demonstrated a much higher proportion of GFP-expressing cells compared to those transfected with B7 (Fig. 5).

**Figure 6:**
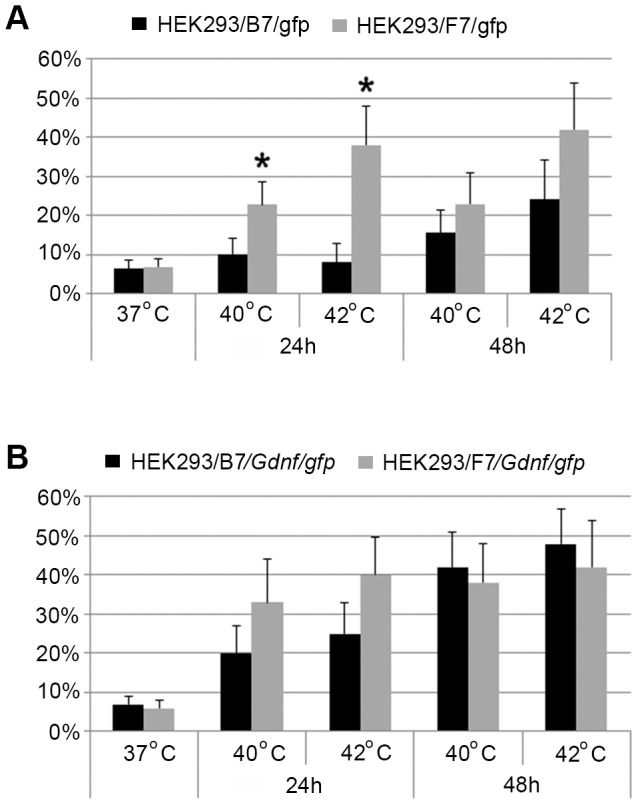
Heat shock effect on HEK293/B7/*gfp* and HEK293/F7/*gfp* (A) and HEK293/B7/*Gdnf/gfp* and HEK293/F7/*Gdnf/gfp* (B) cells studied by flow cytometry. A. Flow cytometry data on HEK293/B7/*gfp* and HEK293/F7/*gfp* cells 24h and 48 h after heat shock at 40 and 42°C. The proportion of GFP-positive HEK293/F7/*gfp* cells 24 and 48 h after heat shock was significantly higher than the control at 37°C (p<0.05). A significant increase in the proportion of GFP-positive HEK293/B7/*gfp* cells was observed only 48 h after heat shock (p<0.05). Asterisk indicates significant difference between HEK293/B7/*gfp* and HEK293/F7/*gfp* cells (p<0.05). B. Flow cytometry data on HEK293 cells transfected with B7/*Gdnf-gfp* and F7*Gdnf/gfp*. The same cultures not exposed to heat shock were used as control (37°C). The proportion of GFP-positive cells 24 and 48 hours after heat shock at 40 and 42°C significantly (p<0.05) differed from the control (37°C). Data represent the mean - SD from three independent experiments.

A notable increase in the background GFP expression was observed in cells transfected with F7 but not in those transfected with B7 24 h after heat shock at 40°C. Flow cytometry experiments tested the effect of heat shock at 40 and 42°C 24 and 48 h after heat shock (Fig. 6A). In F7-transfected culture, the proportion of GFP-expressing cells increased to 23% 24 h after heat shock at 40°C and to almost 40% after heat shock at 42°C. The proportion of GFP-expressing cells remained largely unaltered 48 h after heat shock. B7-transfected cell culture demonstrated a substantial increase in the proportion of GFP-expressing cells only 48 h after heat shock at 42°C.

Thus, the *Drosophila hsp70* promoter in the B7 construct with four HSEs is activated at the same temperatures as the *hsp70* promoter in mammalian cells. At the same time, the promoter in the F7 construct with eight HSEs is substantially activated at lower temperatures as well.

**Figure 7:**
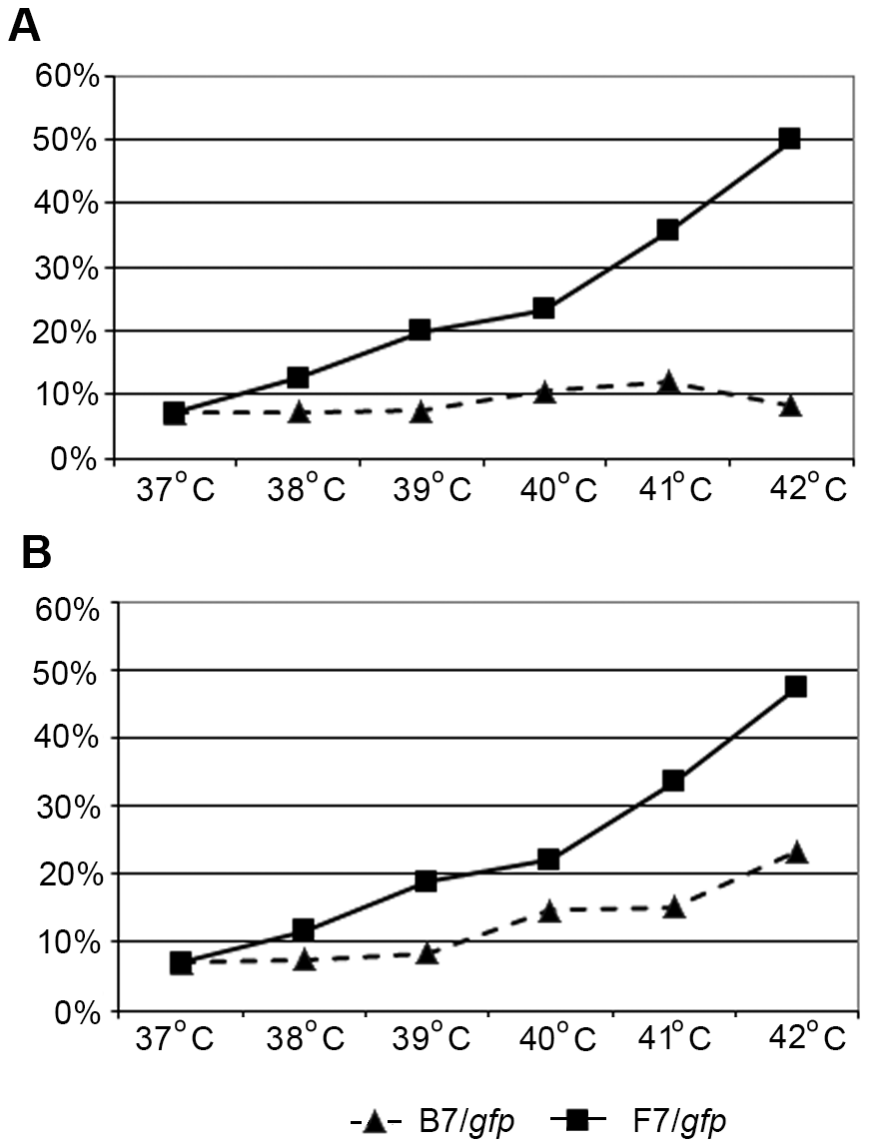
Temperature dependence of *Drosophila* hsp70 promoter activation in HEK293/B7/*gfp* and HEK293/F7/*gfp* cells studied by flow cytometry. Ordinate: proportion of GFP-positive cells 24 h (A) and 48 h (B) after heat shock at 38–42°C and at 37°C (control).

A more thorough investigation of the heat shock effect on the promoter activation demonstrated an increased proportion of GFP-expressing cells transfected with the F7 vector as early as 24 h after heat shock at 38°C and higher (Fig. 7). The cells transfected with B7 demonstrated a similar response only 48 h after heat shock at 40°C and higher.

Preliminary experiments demonstrated that the number of GFP-expressing cells decreases to the background level 96 h after the first heat shock. However, second heat shock reactivated the promoter and the synthesis of the marker protein.

### Constructs Containing glial cell line-derived neurotrophic factor (*Gdnf*) gene under control of drosophila *hsp70* promoter

**Figure 8:**
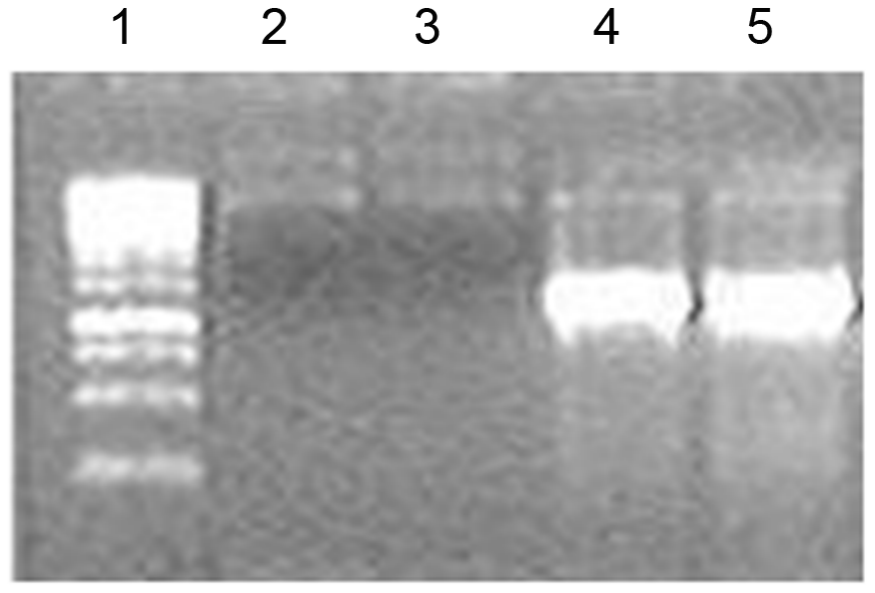
Gel electrophoresis of plasmid DNA amplified by PCR using primers *Gdnf*-F and *gfp*R. 1, 100(Termo Scientific, # SM0244); 2, B7; 3, F7; 4, B7/*Gdnf*; 5, F7/*Gdnf*.

**Figure 9:**
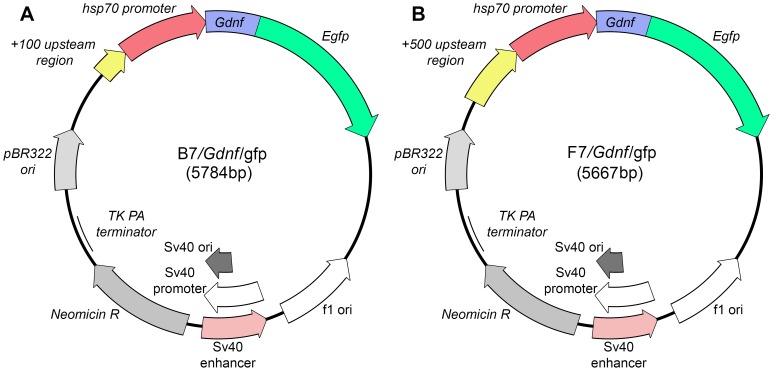
Genetic constructs containing a chimeric gene encoding glial cell line-derived neurotrophic factor and green fluorescent protein under control of hsp70 promoter with different regulatory regions of (A) 100 bp (B7/*Gdnf/gfp*) and (B) 500 bp (F7/*Gdnf/gfp*).

B7 and F7 vectors were used to engineer the constructs containing the *Gdnf* gene under control of *Drosophila hsp70* promoter. PCR fragment (amplified using primers T3 (5^′^-attaaccctcactaaaggga-3^′^) and *Gdnf*-BamHI (5^′^-tggatcccagatacatccacaccttttagcgg-3^′^) was cloned into pGEM-T Easy. The authenticity of the pGEM-T/*Gdnf* construct was confirmed by restriction endonuclease analysis, PCR, and sequencing. The EcoRV-BamHI fragment of the *Gdnf* gene (600 bp) of this plasmid was cloned into SmaI and BamHI sites of B7 and F7. The authenticity of the resulting constructs was confirmed by restriction endonuclease analysis and PCR analysis using primers *Gdnf*-F (5^′^-ggaatcggcaggctgcagctg-3^′^) and *gfp*-R (5^′^-aataaagcttgcatggcggtaatacg-3^′^) (Fig. 8). In addition, they were sequenced using primers *Gdnf*-F (5^′^-ggaatcggcaggctgcagctg-3^′^) and *Gdnf*-R (5^′^-aatgctttcttagaatatggt-3^′^). The diagrams of the genetic constructs are shown in Fig. 9.

**Figure 10:**
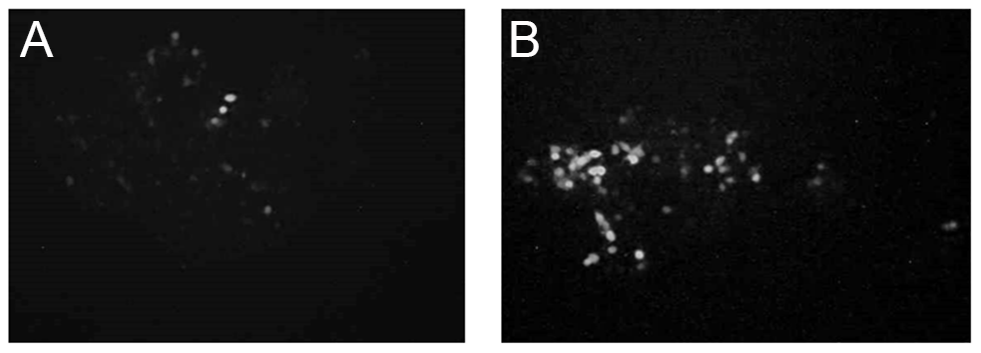
GFP positive HEK293 cells after transfection with B7/*Gdnf/gfp* (A) and F7/*Gdnf/gfp* (B) 24 h after heat shock at 40°C. Scale bar on B – 100 µm.

**Figure 11:**
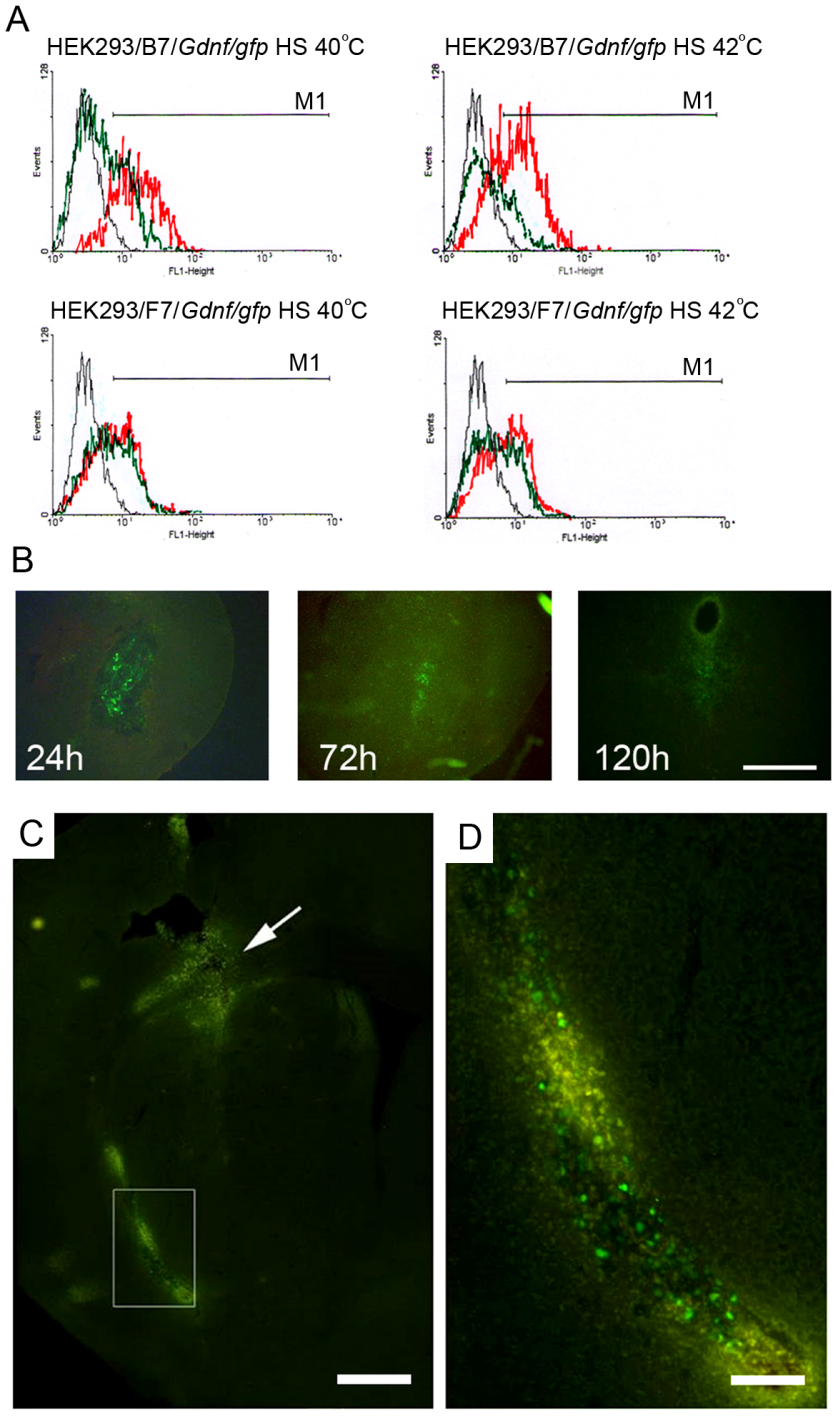
A, Flow cytometry data on HEK293/B7/*Gdnf/gfp* and HEK293/F7/*Gdnf/gfp* cells 24 (red) and 48 h (green) after heat shock at 40 (left) and 42°C (right). B, HEK293 cells transfected with F7/*gdnf-gfp* 24 h, three days (72 h), and five days (120 h) after heat shock at 42°C and injection into the caudate-putamen (striatum) of mouse. Frontal section of the mouse brain (40 µm) at the injection site. Scale bar -0.5 mm. C, Transgenic GFP expression in not heat-shocked HEK293/F7/gfp cells in the mouse brain three days after injection of cells and endothelin-1 (indicated by the arrow) and at a distance from the injection site (the fraimed area in A is enlarged in B). Scale bar on C is 500 µm and on D 100 µm.

Isolated and purified B7/*Gdnf/gfp* and F7/*Gdnf/gfp* were introduced into HEK293 cells by transfection. After 24 h of culture, transfected cells were exposed to heat shock at 40 or 42°C for 60 min. GFP expression was studied using fluorescent microscope Olympus IX81 with a blue excitation filter and a yellow blocking filter (Fig.10) and flow cytometry (Fig. 11 A).

GFP expression was observed in both cultures 24 h after the exposure to heat shock at 40°C. A fraction of studied cells exposed to heat shock was fixed with 4% formaldehyde to evaluate GFP fluorescence using flow cytometry on a FACScan (Fig. 11 A). The flow cytometry data demonstrated fluorescence of the GDNF/GFP fusion protein 24 h after heat shock at 40°C. Note that the effect of HSE number on the promoter induction was not very pronounced: four HSEs, 30.3% fluorescent cells; eight HSEs, 43% fluorescent cells. However, the proportion of fluorescent cells notably increased 48 h after heat shock. Cells transfected with B7/*Gdnf/gfp* (with four HSEs) demonstrated a substantial increase in the proportion of fluorescent cells: from 30.3 to 82.2%. The temperature of 42°C proved less efficient for the induction of GDNF/GFP expression in the case of both four and eight HSEs. However, the difference leveled off 48 h after heat shock (51% in the case of 40°C and 53% in the case of 42°C) (Fig. 6B).

GFP expression decreases 72 h after heat shock. However, repeated heat shock reactivates the protein expression.

### Behavior of Transfected HEK293 Cells in Mouse Brain

Two series of experiments were carried out to study the behavior of cells transfected with B7 and F7. In the first series, HEK293 cells transfected with GFP-expressing B7 and F7 vectors with the regulated *Drosophila* hsp70 promoter were exposed to heat shock at 42°C for 60 min and transplanted into the mouse striatum. After 1, 3, 5, and 10 days, GFP-positive cells were visualized on the sections of the brain in the area of transplantation under a fluorescent microscope (Fig. 11B). The number of GFP-positive cells peaked 24 h after injection and decreased with time. No fluorescent cells were found on the mouse stratum sections 10 days after transplantation. No significant differences in the percentage of cells positive for GFP) have been revealed between cells transfected with B7/*Gdnf/gfp* and F7/*Gdnf/gfp*.

In the second series, transgenic HEK293/B7/*gfp* and HEK293/F7/*gfp* cells were introduced into the brain of mouse with experimental focal cerebral ischemia induced by endothelin-1. Immediately after the injection of this vasoconstrictor, a suspension of about 100,000 cells in 1 µl of Hanks solution was injected to the same site and depth. Prior to injection, the cells were not exposed to heat shock. In control animals the cells were injected without preliminary endothelin-1 administration.

**Figure 12:**
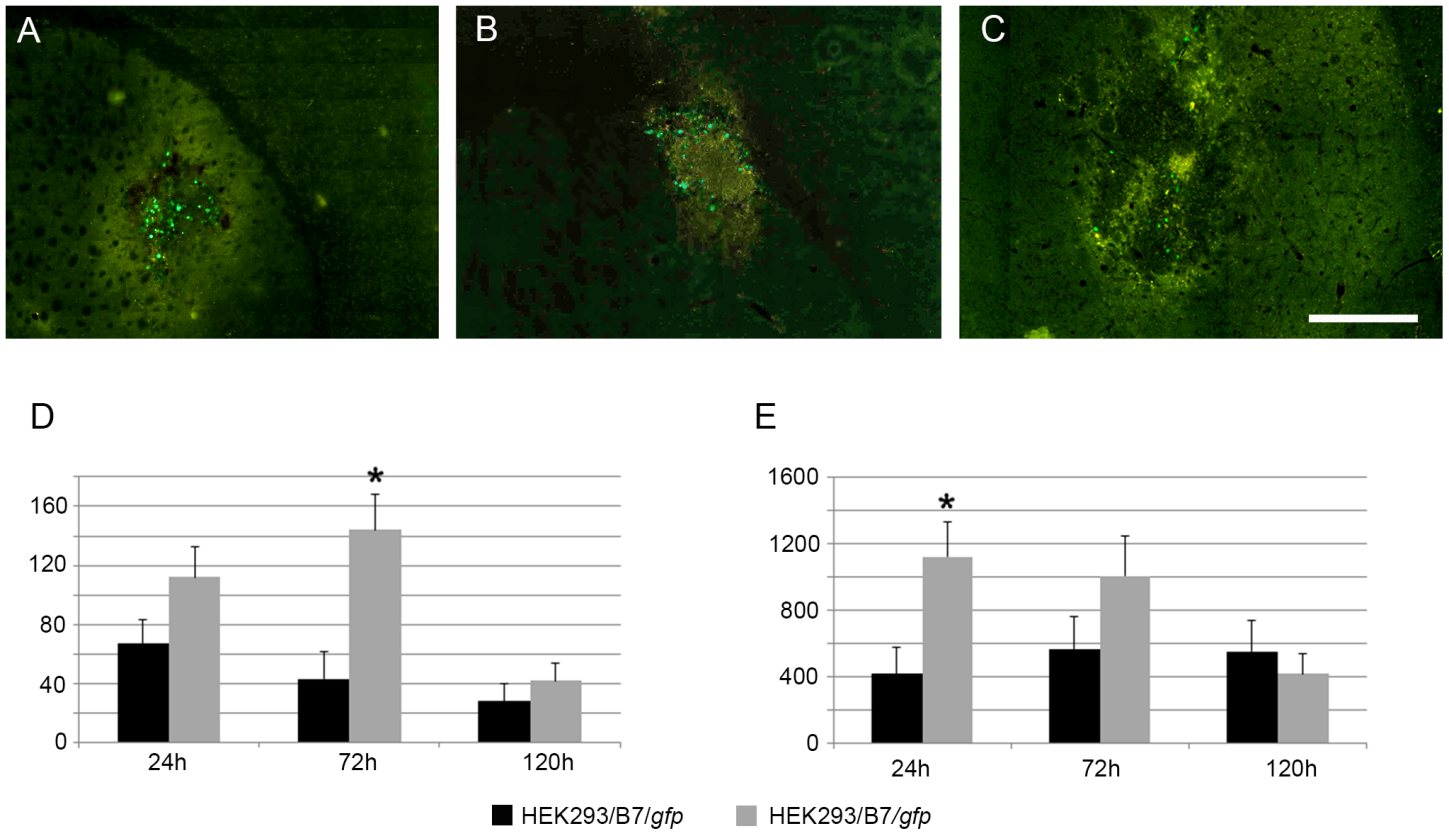
GFP-expression in not heat-shocked HEK293/B7/gfp and HEK293/F7/gfp cells 24, 72, and 120 h after their injection into the mouse brain with and without preliminary endothelin-1 administration. A–C, transgenic GFP expression in not heat-shocked HEK293/F7/gfp cells in the mouse brain three days after injection of cells and endothelin-1 24 h (A), three. days (B), and 5 days (C) after injection of cells and endothelin-1. Scale bar on C–0.5 mm. D, and E, - analysis of GFP-positive HEK293/B7/*gfp* and HEK293/F7/*gfp* cell counts 24, 72, and 120 h after their injection into the mouse brain without preliminary heat shock. GFP positive cells counts found on the brain sections of control animals injected with transfected cells without (C) and with (D) preliminary endothelin-1 administration. Cells were counted using fluorescent microscope on brain sections in the injection area. Cell counts on B were significantly (p<0.05) enhanced in comparison with cell counts on A. Asterisk indicates significant difference (p<0.05) between cells transfected with B7/*gfp* and F7/*gfp*.

These experiments demonstrated numerous transgenic cells in the injection area 24 h after transplantation. Only single GFP-positive cells were observed in the injection area 3 days later. Much more GFP-positive cells were found outside the injection area. Most cells were near vessels or the cerebral white matter (Fig. 11C, D). A minor fraction of cells were in the first layer of the cerebral cortex. Five days after the injection, only small numbers of GFP-positive cells could be found on brain sections in the injection area. Only individual cells (1–2 per section) could be found on the brain sections of control animals injected with transfected cells without preliminary endothelin-1 administration (Fig. 12).

## Discussion

As demonstrated previously, *D. melanogaster hsp70* promoter introduced into mammalian cells can function as a temperature-dependent promoter [18], [19]. In this work, cells were exposed to 45°C to activate the promoter. Schweinfest used transgenic mammalian cells expressing c-myc under control of the *D. melanogaster hsp70* to demonstrate the activation of the promoter and transgene expression at 42°C [20]. Our experiments [8] showed that the temperature of *hsp70* promoter activation can be decreased to 37°C if the same cells were transfected with a construct containing the HSP90 gene under control of the CMV promoter. Hence, there are mechanisms that can be used to decrease the temperature threshold for *D. melanogaster hsp70* promoter activation. Andreeva with coauthors demonstrated that *Drosophila hsp70* promoter activity does not necessarily correspond to the heat shock temperature of transfected species [21]. For instance, the transfection of a construct with *Drosophila hsp70* promoter into loach embryonic cells led to the constitutive expression of promoter-controlled GFP irrespective of culture temperature. Thus, temperature regulation of *Drosophila hsp70* promoter depends on the regulatory proteins of the host cell. Our experiments demonstrated that the number of HSEs at the 5′-end of the *Drosophila hsp70* promoter modulates its sensitivity and activation duration. We have elected to use four and eight HSEs considering that the promoter regions of *hsp70* in *D. virilis* and *D. lummei* contain two HSEs each, while the *D. melanogaster* promoter has four HSEs [14], [22], [23]. At the same time, the human heat shock promoter contains 1 to 3 HSEs [24], [25], [26]. Commonly there are two HSEs in humans, and HSE number increased by one improves the promoter response to heat shock. Our experiments with B7 and F7 vectors containing four and eight HSEs at the 5′-end of the *Drosophila* promoter, respectively, demonstrated the F7 promoter activation already at 38°C in human cells (Fig. 6), and its activity in this construct at higher temperatures was much higher compared to B7. HSE number increased to eight not only significantly improved the activation of the *D. melanogaster* hsp70 promoter but also shortened the period before reaching the peak promoter activity after heat shock. In the case of four HSEs at the 5′-end of the promoter, its activity peaked 48 h after heat shock and this peak was half that of the promoter with eight HSEs; moreover, the latter was already observed after 24 h and is maintained for 48 h (Fig. 6).

Transplantation of transgenic HEK293/B7 and HEK293/F7 cells into the mouse brain parenchyma demonstrated promoter activation and long-term GFP expression in the ischemic focus without heat shock prior to transplantation. Thus, both constructs can be activated by heat shock and likely by inflammatory processes in the brain ischemic focus, where GFP expression was observed for at least three days.

Further investigation of the activity of constructs used in this work should determine the applicability of *Drosophila hsp70* promoter combined with different number of HSEs in the upstream region as a regulated promoter in genetic therapeutic systems.
